# MPP2 is a postsynaptic MAGUK scaffold protein that links SynCAM1 cell adhesion molecules to core components of the postsynaptic density

**DOI:** 10.1038/srep35283

**Published:** 2016-10-19

**Authors:** Nils Rademacher, Bettina Schmerl, Jennifer A. Lardong, Markus C. Wahl, Sarah A. Shoichet

**Affiliations:** 1 Neuroscience Research Center and Cluster of Excellence NeuroCure, Charité-Universitätsmedizin Berlin, Charitéplatz 1, 10117 Berlin, Germany; 2 Institute of Chemistry and Biochemistry, Structural Biochemistry, Freie Universität Berlin, Takustr. 6, 14195 Berlin, Germany

## Abstract

At neuronal synapses, multiprotein complexes of trans-synaptic adhesion molecules, scaffold proteins and neurotransmitter receptors assemble to essential building blocks required for synapse formation and maintenance. Here we describe a novel role for the membrane-associated guanylate kinase (MAGUK) protein MPP2 (MAGUK p55 subfamily member 2) at synapses of rat central neurons. Through interactions mediated by its C-terminal SH3-GK domain module, MPP2 binds to the abundant postsynaptic scaffold proteins PSD-95 and GKAP and localises to postsynaptic sites in hippocampal neurons. MPP2 also colocalises with the synaptic adhesion molecule SynCAM1. We demonstrate that the SynCAM1 C-terminus interacts directly with the MPP2 PDZ domain and that MPP2 does not interact in this manner with other highly abundant postsynaptic transmembrane proteins. Our results highlight a previously unexplored role for MPP2 at postsynaptic sites as a scaffold that links SynCAM1 cell adhesion molecules to core proteins of the postsynaptic density.

Scaffold proteins are structural proteins that are essential for the assembly of functional protein complexes. They include a diverse group of molecules, most of which are large multi-domain proteins that can bind various interactors simultaneously. We are interested in the membrane-associated guanylate kinase (MAGUK) scaffold proteins, which share the common ‘MAGUK core domains’ (PDZ, SH3, and GK). MAGUKs are critically important in multiple tissues for the formation of multiprotein complexes involved in cell-cell communication; e.g. they are involved in tight junction formation^1^,^2^, and several MAGUK family members exhibit high expression in neurons, where they regulate formation and maintenance of synapses^3^,^4^,^5^.

At synapses of central neurons, the importance of scaffold proteins is exemplified in the postsynaptic density (PSD), a highly organised structure consisting of multiple densely packed interacting proteins (for review see ref. 6). Some members of the MAGUK family, namely the PSD-95 family proteins, have been described and characterised in detail with regard to their functions at the PSD. Together with other neuronal scaffold proteins, they provide a structural framework and thereby facilitate regulation of synaptic transmission. In addition to serving as central interaction hubs for cytosolic proteins that are situated within the postsynapse^7^, PSD-95 and related family members are particularly important for synaptic targeting and regulated trafficking of neurotransmitter receptors^8^,^9^. This established function of PSD-95 relies on PDZ-ligand interactions between the N-terminal PDZ domains of PSD-95 and the PDZ-binding C-termini of specific receptor subunits^10^. Structurally related MAGUKs SAP102, PSD-93, and SAP97 are likewise capable of influencing the receptor content of glutamatergic synapses; together these molecules are critically important for regulating fast synaptic transmission in the mammalian brain.

MAGUK scaffold proteins have also been implicated in cell-cell communication via direct, PDZ domain-mediated interactions with cell adhesion molecules. For example, the formation of tight junctions in epithelial cells relies on both the interaction between the extracellular domains of transmembrane cell adhesion molecules such as claudins and occludins, and the presence of the cytosolic zonula occludens (ZO) subfamily of MAGUK scaffolds^11^. The MPP MAGUK family proteins (MAGUK p55 subfamily members 1-7) are also important for organising protein complexes at such intercellular junctions via PDZ domain interactions^12^,^13^. At synapses, direct cell-cell contacts are likewise orchestrated via extracellular interactions between cell adhesion molecules. Trans-synaptic adhesion complexes align pre- and postsynaptic membranes by interacting with their extracellular domains across the synaptic cleft. Various synaptic adhesion molecule pairs have been characterised and their importance for synapse maturation and function is recognised^14^.

As in other cell types, these neuronal cell adhesion molecules bind to intracellular scaffold molecules via PDZ domain interactions, and it has been demonstrated that MAGUKs can likewise serve this function in neurons, as they do in epithelial cells. At the presynapse, the MAGUK protein CASK is well-known for its PDZ-mediated interaction with the presynaptic cell adhesion molecule Neurexin^15^. At the postsynaptic side, the prototypical synaptic MAGUK scaffold PSD-95 serves this function for a set of postsynaptic cell adhesion molecules, including e.g. Neuroligin^16^, SALM^17^, and NGL^18^, but for several other trans-synaptic cell adhesion molecules (e.g. Nectin-1, SynCAMs, LRRTMs), the link to postsynaptic structures is less clear.

While the MPP family of MAGUK proteins is best known for its scaffolding function in epithelial cells, more recent studies in *Drosophila* suggest a putative role in neurons as a postsynaptic scaffold^19^. In addition, high-throughput studies aimed at the identification of novel members of glutamate receptor protein complexes highlighted a possible role for mammalian MPP2 at synapses^20^. We therefore explored the idea that MPP2 might be a novel synaptic MAGUK protein. Here we show that MPP2 is indeed a postsynaptic protein expressed in mammalian neurons. We also show that it binds to GKAP and PSD-95, two major components of the postsynaptic density of mammalian glutamatergic synapses. We demonstrate that MPP2 colocalises with the synaptic cell adhesion molecule SynCAM1 (also sometimes referred to as CADM1, TSLC1, or Igsf4) and directly binds SynCAM1 via its PDZ domain, and thus highlight a novel structural link between the SynCAM1 cell adhesion complex and several core components of the PSD.

## Results

### MPP2 is a synaptic MAGUK protein

**Figure 1:**
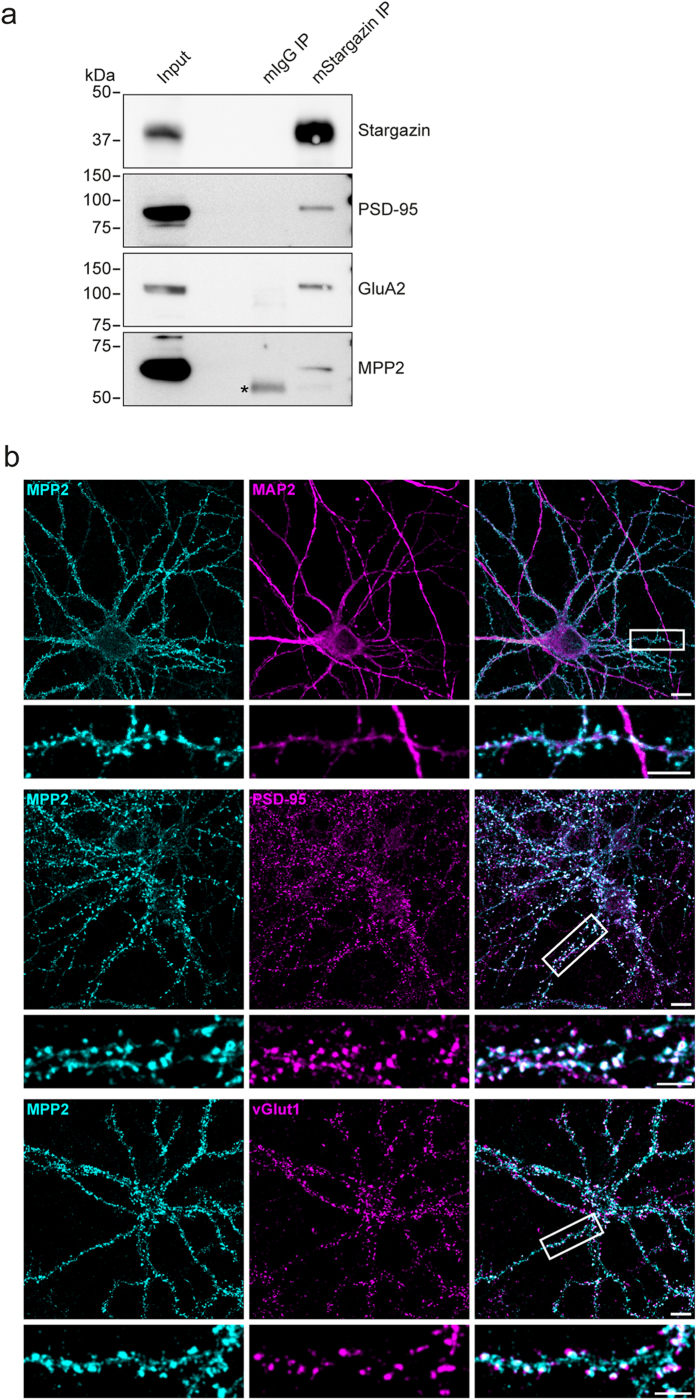
MPP2 is a component of postsynaptic receptor complexes. (**a**) The AMPA receptor auxiliary subunit Stargazin/Tarpγ2 was immunoprecipitated from crude synaptosomal preparations of adult rat brain with a monoclonal mouse antibody and precipitates were analysed by western blot, with antibodies to synaptic proteins, as indicated. A pull-down with mouse IgGs (mIgG IP) was performed as negative control (a non-specific IgG background band is marked with a*). For uncropped blots, see Supplementary Fig. S2. (**b**) Cultured rat hippocampal neurons (E18) were fixed at DIV28 and stained for MPP2 (left panels) together with the microtubule-associated protein 2 (MAP2, dendritic marker), the postsynaptic density protein 95 (PSD-95, postsynaptic marker) or the vesicular glutamate transporter 1 (vGlut1, presynaptic marker) and respective secondary antibodies. Overlapping signals are visible in the merged images on the right, with the indicated regions selected for detailed images. Scale bars: overview = 10 μm, detail = 5 μm.

In order to explore the possibility that MPP2 might be an important member of synaptic protein complexes, we examined the content of AMPA receptor complexes using immunoprecipitation. Following solubilisation of membranes from rat brain crude synaptosomal preparations, we pulled down the well-established AMPA receptor auxiliary subunit Stargazin/TARPγ2 using a monoclonal mouse antibody and analysed the associated proteins by SDS-PAGE and western blot in a targeted approach. As expected, we identified the AMPA receptor subunit GluA2 and the synaptic MAGUK scaffold protein PSD-95, both of which are established direct binding partners for the TARP family of AMPA receptor auxiliary subunits. We also clearly detected MPP2 in these immunopurified AMPA receptor complexes (see Fig. 1a), indicating that this protein is indeed a molecular constituent of AMPA receptor complexes. Importantly, the polyclonal rabbit αMPP2 antibody used for these experiments was first tested in mock- vs MPP2-transfected COS7 cell lysates (see Supplementary Fig. S1a).

We next examined the subcellular MPP2 localisation in neurons. We immunostained mixed cultures of dissociated cells from rat hippocampi with antibodies against MPP2 and co-stained for the neuronal/dendritic marker MAP2, the postsynaptic marker PSD-95 and the presynaptic marker vGlut1 (Fig. 1b). In neurons, MPP2 staining could be exclusively detected in cells that were positive for MAP2, confirming its presence in neurons (Fig. 1b, upper panel). Further analysis revealed that the endogenous MPP2 signal overlaps with PSD-95 staining, indicating a postsynaptic localisation of MPP2 (Fig. 1b, middle panel). This finding is strongly supported by the observed adjacent MPP2 staining relative to the presynaptic vGlut1 staining (Fig. 1b, lower panel). Importantly, we also ensured that this polyclonal rabbit αMPP2 antibody indeed detects MPP2 in immunofluorescence stainings via control experiments with recombinant MPP2 (see Supplementary Fig. S1b).

### MPP2 interacts with the postsynaptic density proteins PSD-95 and GKAP

**Figure 2:**
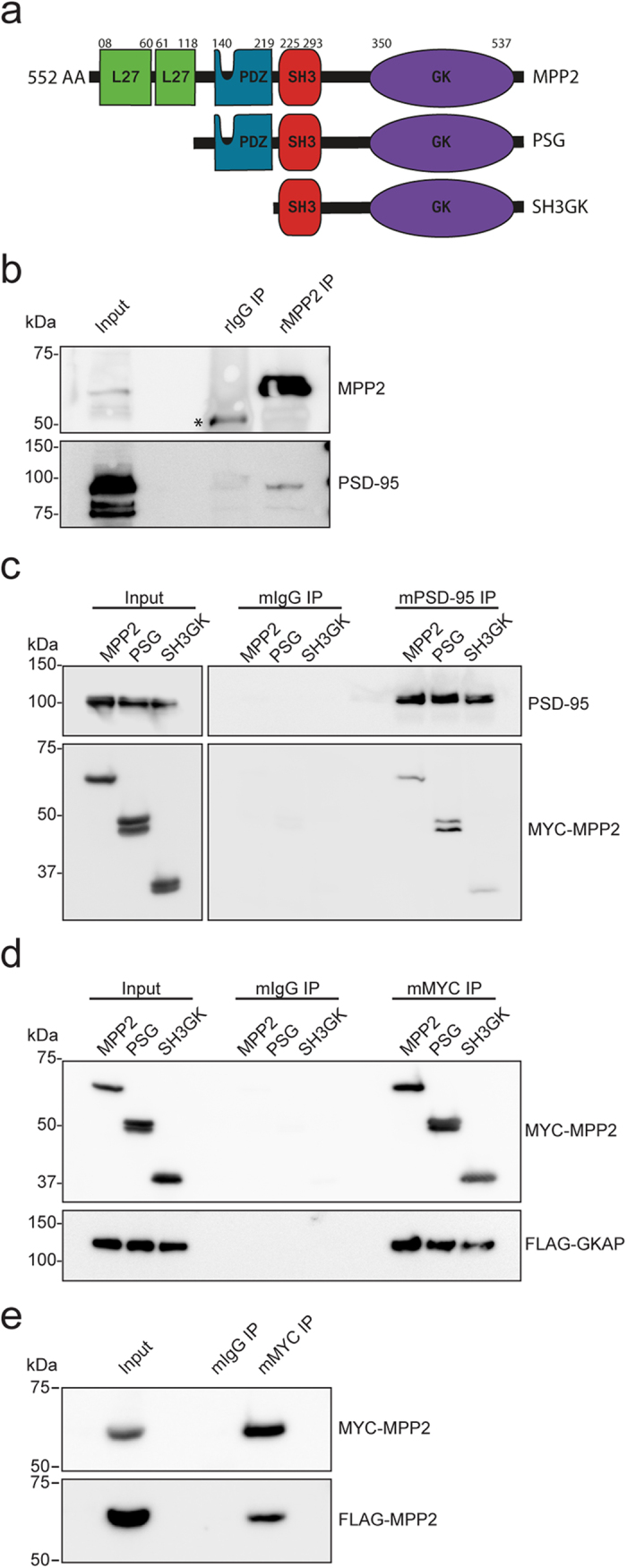
MPP2 interacts with the postsynaptic density proteins PSD-95 and GKAP, and it homomultimerises. (**a**) Structure of the murine membrane-associated guanylate kinase protein MPP2 and corresponding deletion constructs used in this study. L27 domains (green), PDZ domain (blue) and SH3-GK tandem domain (red, purple). (**b**) Immunoprecipitation of MPP2 (using a rabbit polyclonal MPP2 antibody or rabbit IgGs as a negative control) from a crude synaptosome preparation of adult rat brain coprecipitates PSD-95, as detected by western blot with antibodies to PSD-95 (coIP) or MPP2 (IP control). A background IgG signal is marked with a*. (**c**) MYC-tagged MPP2 (MPP2), MYC-tagged MPP2 PDZ-SH3GK (PSG), or MYC-tagged MPP2 SH3GK (SH3GK) were coexpressed in COS7 cells with recombinant PSD-95 and immunoprecipitated with αPSD-95 antibody (mPSD-95 IP) or with mouse IgGs (mIgG IP) as a negative control. Coprecipitated proteins were analysed by western blot with αMYC antibody, pulldown controls with a PSD-95 antibody. (**d**) MYC-tagged MPP2 (MPP2), MYC-tagged MPP2 PDZ-SH3GK (PSG), or MYC-tagged MPP2 SH3GK (SH3GK) were coexpressed with FLAG-tagged recombinant GKAP in COS7 cells and immunoprecipitated with αMYC antibody (mMYC IP) or with mouse IgGs (mIgG IP) as a negative control. Coprecipitated proteins were analysed by western blot with αFLAG antibody, pulldown controls with αMYC antibody. (**e**) FLAG- and MYC-tagged MPP2 proteins were coexpressed in COS7 cells. MYC-MPP2 was pulled down with αMYC antibody (mMYC IP) and coprecipitated FLAG-tagged MPP2 was analysed by western blot with αFLAG antibody. Mouse IgGs (mIgG IP) served as a negative control. For uncropped blots, see Supplementary Fig. S2.

The MAGUK family proteins in general are membrane-associated scaffold proteins that regulate assembly of protein complexes. MPP2 contains two N-terminal L27 domains which are known to be involved in heterodimerisation, followed by the MAGUK signature domain module PDZ-SH3-GK (see Fig. 2a). Since we could copurify MPP2 and PSD-95 in a protein complex that is associated with AMPA receptors (Fig. 1a), and we observed colocalisation of MPP2 and PSD-95 at postsynaptic sites in hippocampal neurons (Fig. 1b), we explored the interaction between MPP2 and PSD-95 in more detail. We first immunoprecipitated endogenous MPP2 from rat brain crude synaptosomes and could clearly identify PSD-95 in the precipitate (Fig. 2b). Additionally, recombinant MYC-MPP2, MYC-PDZ-SH3-GK (MPP2 PSG module) and MYC-SH3-GK coimmunoprecipitated with PSD-95 following expression in heterologous cells (Fig. 2c), indicating that MPP2 is indeed capable of binding PSD-95 independent of its PDZ and L27 domains, which supports the idea that these proteins interact via cytosolic scaffold interactions, rather than indirectly via their PDZ-mediated associations with transmembrane proteins. Indeed, both of these proteins have the potential to bind multiple cytosolic proteins via their C-terminal interaction domains, and these scaffolding properties of PSD-95 have been studied in detail.

The idea that MPP2 also acts as a synaptic scaffold molecule, however, is a new one that has not been explored in depth. In a yeast two-hybrid screen of a rat hippocampus cDNA library we also identified GKAP (guanylate kinase associated protein; also known as SAPAP1 and DLGAP1), an established synaptic structural protein, as a second putative direct interactor of MPP2 (see Supplementary Table S1 for Y2H results). We confirmed that both the MPP2 PSG module (used as bait in the Y2H screen) and full-length MPP2 interacted with GKAP via coimmunoprecipitation following expression of tagged recombinant proteins in heterologous cells (Fig. 2d). We also coprecipitated GKAP with the isolated SH3-GK sub-module of MPP2, indicating that the interaction with GKAP is mediated by these domains in MPP2, which is in line with studies on other MAGUKs suggesting that the GK domain is a non-functional kinase domain that has evolved to play an important role in protein-protein interactions^7^.

### MPP2 homomultimerises

In addition to its role within the PSD-95-MPP2-GKAP scaffold complex, we explored the idea that MPP2 molecules might be capable of direct intermolecular interactions, as has been predicted, e.g., for other MPP family members^21^. Indeed, following expression of MPP2 recombinant proteins, we were able to coprecipitate differentially tagged variants: after pull-down with MYC-tagged MPP2, we observed FLAG-tagged MPP2 in coprecipitates (Fig. 2e), suggesting that MPP2 proteins are capable of intermolecular interactions to form dimers or oligomers.

### MPP2 is a SynCAM1 interactor

**Figure 3:**
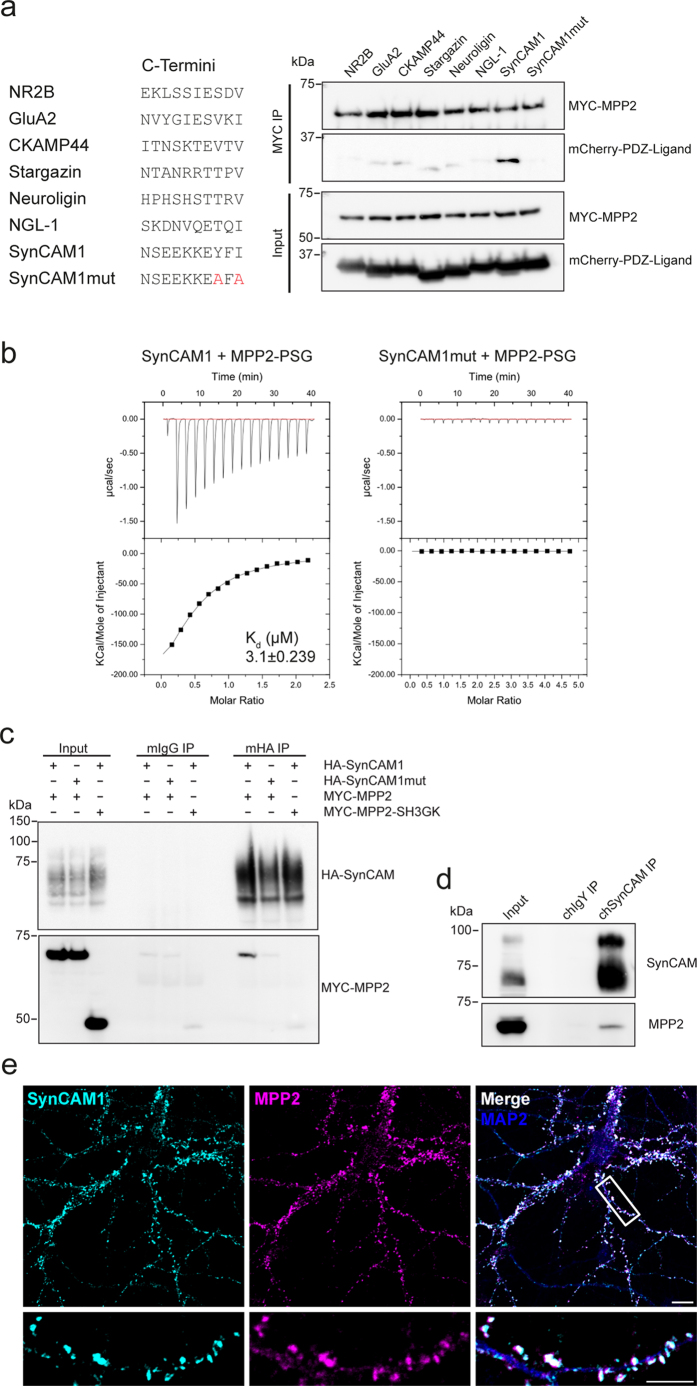
MPP2 binds directly to the synaptic adhesion molecule SynCAM1. (**a**) Targeted identification of putative MPP2 PDZ domain interactors: The 10 C-terminal amino acids of known synaptic PDZ-ligand proteins (see table on the left) were fused to the monomeric mCherry tag and tested for interaction with MYC-tagged MPP2 in coimmunoprecipitation experiments. Pulldown control (MYC-MPP2) and coprecipitated proteins (mCherry-PDZ-Ligand) were detected by western blot (upper panels); input controls are shown below. For uncropped blots, see Supplementary Fig. S3. (**b**) Isothermal titration calorimetry measurements with 350 μM mCherry-SynCAM1 and SynCAM1mut, respectively, injected to 55 μM MPP2-PSG module revealed a robust binding of the SynCAM1 C-terminus with a K_d_ of 3.1 ± 0.239 μM (left panels). Results for the mutated SynCAM1 C-terminus control are shown on the right. (**c**) Full-length HA-tagged SynCAM1 (HA-SynCAM1) and MYC-tagged MPP2 (MYC-MPP2), HA-tagged mutant SynCAM1 (HA-SynCAM1mut) and MYC-MPP2, or HA-SynCAM1 and MYC-tagged MPP2 SH3-GK (MYC-MPP2-SH3GK) were coexpressed in COS7 cells and immunoprecipitated with αHA (mHA IP) or mouse IgGs (mIgG IP) as a negative control. Pulldown controls are shown (HA-SynCAM); coprecipitated proteins are detected by western blot with αMYC antibody below. For uncropped blots, see Supplementary Fig. S3. (**d**) Immunoprecipitation of SynCAM1 (using a chicken SynCAM1-Biotin antibody or chicken IgYs as a negative control) from a crude synaptosome preparation of adult rat brain coprecipitates MPP2, as detected by western blot with antibodies to MPP2 (coIP) or SynCAM1 (IP control).(**e**) Cultured rat hippocampal neurons (E18) were fixed at DIV21 and stained for endogenous SynCAM1, MPP2 and MAP2 (depicted together with merged image on the right). Region selected for detailed image is indicated; scale bars: overview = 10 μm, detail = 5 μm.

To date, most studies on MPP2 have centered on its role in epithelial cells, and little is known about its role in neurons. We have demonstrated that MPP2 is a postsynaptic MAGUK scaffold: it interacts with the established synaptic proteins PSD-95 and GKAP, and it is present in Stargazin/AMPA receptor coprecipitates from rat synaptosomes. As this finding is new, a search for the synaptic PDZ domain ligands that bind MPP2 has not been carried out to date. We therefore used a targeted approach to test a set of established synaptic C-termini that are known to bind PDZ domains. Following coexpression of MPP2 with the most abundant synaptic C-termini (see table in Fig. 3a for protein names and C-terminal amino acid sequences) and subsequent pull-down of MPP2, we observed no coprecipitation of NR2B, GluA2, CKAMP44, Stargazin/TARPγ2, Neuroligin, or NGL-1 (Fig. 3a blot). However, using the same strategy, we identified the brain-specific synaptic cell adhesion molecule SynCAM1 C-terminus as a potential MPP2 interactor (Fig. 3a). Among PDZ ligands tested, only the SynCAM1 C-terminus efficiently bound to MPP2; moreover, while the wild-type SynCAM1 C-terminus was significantly enriched in the MPP2 precipitate, a mutant SynCAM1 C-terminus (harbouring point mutations at positions 0 and -2) did not bind to MPP2 (Fig. 3a), confirming specificity of the interaction for the C-terminal PDZ-binding motif. SynCAM1 exhibits a class 2 C-terminal PDZ ligand sequence that fundamentally differs from the other C-termini tested in that it harbours large aromatic residues at the -1 (phenylalanine) and -2 (tyrosine) positions. A direct interaction between MPP2 and SynCAM1 was supported by the results of our Y2H screen (see Supplementary Table S1). We also used bacterially produced MPP2 PDZ-SH3-GK (MPP2 PSG module) and mCherry-tagged SynCAM1 (or SynCAM1 mutant) proteins to test for a direct interaction between the SynCAM1 C-terminus and the MPP2 PSG module via isothermal titration calorimetry. The MPP2 PSG module binds to the SynCAM1 construct with a K_d_ of 3.1 μM, whereas the SynCAM1mut construct with alanine substitutions at positions 0 and -2 of the PDZ ligand sequence did not bind at all (Fig. 3b). An MPP2-SynCAM1 interaction could also be confirmed by pull-down experiments of full-length HA-tagged SynCAM1 with full-length MYC-tagged MPP2 expressed in heterologous cells, whereas the full-length HA-SynCAM1mut construct did not interact with MPP2 in the comparable coIP experiment. Importantly, an MPP2 construct consisting exclusively of the SH3-GK domains did not copurify with full-length SynCAM1 (Fig. 3c), indicating that binding is indeed mediated by the PDZ domain. These findings are further supported by experiments with endogenous proteins purified from rat brain crude synaptosomes. Following immunoprecipitation of SynCAM1, MPP2 is enriched in the precipitate (Fig. 3d).

**Figure 4:**
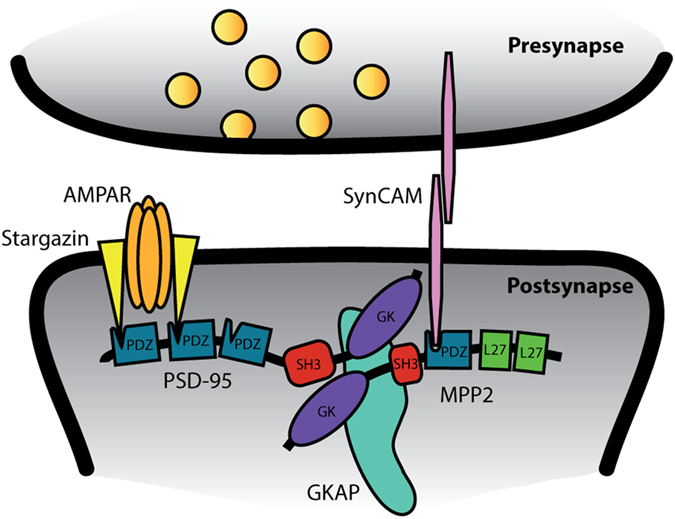
A model illustrating postsynaptic MPP2 protein interactions. Our data indicate that MPP2 is a novel postsynaptic scaffold protein that binds to the C-terminus of the trans-synaptic SynCAM1 (pink) via its PDZ domain (blue). By interaction of the MPP2 SH3-GK domain (red, purple) with PSD-95, MPP2 links SynCAM1 to postsynaptic AMPA receptor complexes. The SH3-GK domain of MPP2 also interacts with GKAP (turquoise), thereby providing a link between MPP2 and deeper postsynaptic scaffolds.

Upon analysis of endogenous proteins in cultured primary hippocampal neurons, we observed a clear overlap of SynCAM1 and MPP2 immunofluorescence signals (Fig. 3e), supporting the idea that SynCAM1 and MPP2 are indeed important binding partners at postsynaptic sites in glutamatergic neurons. Together our data support the idea that MPP2 serves as a new synaptic scaffold that links SynCAM1 to core proteins of the postsynaptic density. Our observations are summarised as a graphical model in Fig. 4.

## Discussion

MPP2 belongs to a group of seven proteins homologous to the *Drosophila* stardust protein, which is a regulator of epithelial cell polarity^12^. Also in vertebrate studies, the functional characterisation of MPP family members has so far centered on their role at epithelial cell junctions. Our results highlight a novel role for MPP2 at synapses: MPP2 is expressed in hippocampal neurons at postsynaptic sites where it links the SynCAM1 cell adhesion molecule via PDZ-ligand interactions to the core postsynaptic density proteins PSD-95 and GKAP.

Given that MPP2 associates with AMPA receptors via interactions with central scaffold proteins of the PSD, one could speculate that alterations in MPP2 expression might influence AMPA receptor synaptic expression and function. Indeed, numerous studies highlight that acute alterations of synaptic MAGUKs can have dramatic effects on AMPA receptor-mediated currents and LTP (e.g. see refs 9,22,23,24). However, unlike PSD-95 and related MAGUKs, MPP2 represents a novel type of synaptic MAGUK in that it does not interact directly with AMPA receptor subunits or auxiliary subunits.

All MPP family proteins contain a single PDZ domain, followed by an SH3-GK domain module. Additionally, MPP2 has two L27 domains at its N-terminus that are known to be involved in heterodimerisation with other L27 domain-containing proteins^25^, and may be responsible for the homomultimerisation properties that we observe in MPP2. Recent proteomic studies highlighted a putative role for MPP2 in neurons^20^, and during the course of this study, a functional interaction between MPP2 and potassium channels was described^26^; however, a direct link between MPP2 and associated transmembrane PDZ ligands has not been previously investigated in the context of the synapse. Our findings that MPP2 interacts directly with the C-terminus of SynCAM1 are in line with previous reports that MPP family proteins are able to bind to SynCAM family members in epithelial cells^27^,^28^,^29^ and open new avenues for investigation into the unknown functions of MPP2 at synaptic sites. In addition to their role in other tissues, SynCAMs are known synaptic cell adhesion molecules: through homophilic and heterophilic interactions between their extracellular domains, these molecules form trans-synaptic complexes^30^,^31^, and it has been demonstrated that these complexes are involved in synaptogenesis (for review see ref. 32) and also in synapse stabilisation^33^,^34^.

Importantly, such trans-synaptic adhesion complexes require associated cytosolic scaffolds at pre-and postsynaptic sites in order to execute their functions in synapse formation. PDZ domain interaction partners for SynCAM1 at the presynapse have been explored (*e.g.* CASK and syntenin; see refs 30,35,36). Evidence for an association between SynCAM1 and the MUPP1 PDZ scaffold molecule at postsynaptic sites in cerebellar Purkinje neurons was reported^37^; however, cytosolic postsynaptic PDZ domain proteins that interact with SynCAM1 in hippocampal or cortical neurons have not been identified previously. Moreover, it has been clearly shown that SynCAM1 does not interact directly with the prototypical postsynaptic scaffold molecules of the PSD-95 MAGUK subfamily^30^,^37^.The postsynaptic localisation and functions of SynCAM1 in the hippocampus are the focus of current investigations: of particular interest, SynCAM1 was recently studied in detail using various high resolution microscopy techniques^38^. This study highlighted a predominance of SynCAM1 at postsynaptic sites; moreover, the authors clearly showed that SynCAM1 molecules cluster in regions that define the borders of the PSD in hippocampal neurons, in line with the idea that they play a role in shaping the PSD and modulating synapse size. In light of our results, it is plausible that MPP2 could function as a bridging protein that links these SynCAM1 patches to PSD-95 molecules that are present at the outer regions of the PSD.

Importantly, given the direct interaction of MPP2 with the SynCAM1 C-terminal tail, and the post-synaptic localisation of MPP2, we propose that one primary function of MPP2 is in modulating the synaptic cell adhesion properties of postsynaptic SynCAMs. Interestingly, it has been shown that SynCAM molecules interact with each other in cis (i.e. neighbouring SynCAM molecules in the membrane cluster together), and that this lateral assembly promotes the trans-synaptic interactions that are involved in synapse formation^39^. While it has been shown that the extracellular domains are involved in this lateral assembly^39^, the role of intracellular proteins in this process has not been explored. The identification of MPP2 as a novel postsynaptic intracellular binding partner for SynCAMs, together with our observation that MPP2 is capable of homomultimerising, provide the basis for our current investigations into the functional role of MPP2 at synapses.

In summary, our study highlights MPP2 as a new postsynaptic MAGUK that interacts with the core cytosolic components of the PSD scaffold, including PSD-95 and GKAP. At the same time, it exhibits a unique PDZ ligand binding specificity: unlike PSD-95, MPP2 binds directly to the C-terminus of the synaptic cell adhesion molecule SynCAM1 and thereby has the potential to functionally link this postsynaptic adhesion molecule to receptor complexes via intracellular scaffold interactions.

## Methods

### Primary neuronal cultures

For mixed primary cultures, E18 Wistar pups were decapitated, and hippocampi were isolated and collected in ice-cold DMEM (Lonza). All animals used were handled in accordance with the relevant guidelines and regulations. Protocols were approved by the ‘Landesamt für Gesundheit und Soziales’ (LaGeSo; Regional Office for Health and Social Affairs) in Berlin and animals reported under the permit number T0280/10. The fetal tissue was partially digested (5 min at 37 °C) with Trypsin/EDTA (Lonza) prior to addition of 10% FBS (Biochrom) in DMEM and subsequent washing with DMEM. Tissue was resuspended in neuron culture medium (Neurobasal A supplemented with B27 and 500 μM glutamine) and mechanically dissociated. Neurons were then plated at ~10^5^ cells/cm^2^ on coverslips coated with poly-D-Lysine and Laminin (both Sigma). One hour after plating, cell debris was removed and cultures maintained for 3–5 weeks in a humidified incubator at 37 °C with 5% CO_2_.

### Constructs

Full-length mouse MPP2 (NM_016695.3) was cloned into pCMV-2A and pCMV-3A cloning vectors to generate N-terminal FLAG and MYC-tagged constructs and truncations. Full length rat SynCAM1 (NM_001012201.1) was synthesised by *eurofins* with an HA-tag after the signal peptide and subcloned into the pCMV vector with *NotI* and *SalI* restriction sites.

C-terminal expression constructs for synaptic ligands were generated as previously described^40^. In brief, the monomeric red fluorescent protein (mCherry) was fused to a glycine-serine linker (3x GGGGS) followed by the 10 amino acid C-terminus of the PDZ ligands SynCAM1, NR2B, GluA2, CKAMP44, Stargazin/TARPγ2, NGL-1 or Neuroligin-1. Site directed mutagenesis was used to generate mCherry-SynCAMmut by exchanging SynCAM1 C-terminal amino acids 0 and −2 to alanines.

### Antibodies

#### For western blot/co-IP

Primary antibodies: αSynCAM-Biotin (chicken, CM004-6, MBL), αSynCAM (rabbit, PA3-16744, Thermo Scientific), αMPP2 (rabbit, ab97290, Abcam), αPSD-95 (rabbit, ab76115, Abcam), αPSD95 (mouse, 75-028, NeuroMab), αGluA2 (mouse, 75-002, NeuroMab), αStargazin/TARPγ2 (mouse, 73-242, NeuroMab) αStargazin (rabbit, 2503S, Cell Signaling), αTubulin (rat, ab6160, Abcam), αFlag-HRP (mouse, A8592, Sigma), αHA (mouse, MMS-101R, Covance), αHA (rabbit, H6928, Sigma), αMyc (mouse, 631206, Clontech), αMYC (rabbit, 2272S, Cell Signalling), αHSV (rabbit, ab3414, Abcam), αGFP (goat, ab6663, Abcam), normal mouse IgG (sc-2025, Santa Cruz), normal rabbit IgG (sc-2027, Santa Cruz), normal chicken IgY (sc-2718, Santa Cruz). Secondary antibodies: αMouse-HRP (115-035-003, Dianova), αRabbit-HRP (111-035-003, Dianova), αRat-HRP (sc-2032, Santa Cruz), αGoat-HRP (sc-2020, Santa Cruz).

#### For immunofluorescence

Primary antibodies: αMPP2 (rabbit, ab97290, Abcam), αMAP2 (mouse, 05-346, Millipore; guinea pig, 188004, Synaptic Systems), αPSD95 (mouse, 75-028, NeuroMab), αvGlut1 (mouse, 75-066, NeuroMab), αSynCAM (chicken, CM004-3, MBL). Secondary antibodies: αMouse Alexa Fluor 405 (A-31553, Invitrogen), αRabbit Alexa Fluor 488 (A-21441, Invitrogen), αChicken Alexa Fluor 488 (703-545-155, Jackson Immuno Research), αMouse Alexa Fluor 568 (A-11031, Life Technologies), αGuinea pig Alexa Fluor 405 (ab175678, Abcam).

### Cell culture and transfection

COS-7 cells were kept in low-glucose DMEM supplemented with 10% FCS, 1000 U/ml penicillin/streptomycin and 2 mM L-glutamine in a humidified incubator at 37 °C with 5% CO_2_. Transfection was performed with Lipofectamine 2000 (Invitrogen) according to the manufacturer’s protocol.

### Coimmunoprecipitation

COS7 cells were transiently transfected with the desired expression constructs as described and harvested 18-20 hrs post-transfection with a cell scraper. Cells were lysed with a 30 gauge syringe needle in immunoprecipitation buffer (50 mM Tris pH 7,4; 100 mM NaCl; 1 mM EDTA, 1% Triton-X or 0.1% NP-40; supplemented with Complete Mini protease inhibitors, Roche). The lysates were cleared by 3x centrifugation at 20000xg. The supernatants were incubated with 2 μg of the appropriate antibody (mouse αMYC (Clontech), mouse αHA (Covance) or normal mouse IgG) with gentle agitation for 3 hours at 4 °C. Pulldown was performed with 30 μl protein-G-agarose beads (Roche) for 1 hour at 4 °C, followed by three washes with IP buffer prior to analysis by western blot.

For brain lysate IPs adult Wistar rats were anaesthetised with isofluorane and sacrificed by decapitation, and reported under permit T0280/10 (LaGeSO); whole brains were removed, rinsed in ice-cold PBS and stored at −80 °C until use. Brains were thawed on ice and homogenised in 20 ml Syn-PER Synaptic Protein Extraction Reagent (Thermo Science) supplemented with Complete Mini protease inhibitors without EDTA (Roche) according to the manufacturer’s protocol. The crude synaptosome fraction was resuspended in immunoprecipitation buffer (50 mM Tris pH 7,4; 100 mM NaCl; 1 mM EDTA, 1% Triton-X; supplemented with Complete Mini protease inhibitors, Roche) and cleared by 3x centrifugation at 20000xg. Immunoprecipitation was performed as described above with 2 μg mouse αStargazin/TARPγ2, mouse αPSD-95 (both NeuroMab) or rabbit αMPP2 (Abcam) antibodies. Endogenous SynCAM was precipitated with 4 μg chicken αSynCAM-Biotin antibody (MBL) and 50 μl Dynabeads M-280 Streptavidin (112.05D, Invitrogen). Normal chicken IgY, mouse IgG or rabbit IgG (all Santa Cruz Biotechnology), respectively, served as negative controls.

### Immunocytochemistry/Immunofluorescence

Immunofluorescence was performed according to standard IF protocols. Primary rat hippocampal neurons were fixed at DIV21-24 and COS7 cells one day after transfection with 4% PFA in PBS for 10 min at RT. Cells were permeabilised with 0.2% Triton-X in PBS for 5 min and blocked for 1 h at RT with blocking solution (4% BSA in PBS). Primary antibodies were incubated overnight at 4 °C diluted 1:500 in blocking solution, followed by incubation with appropriate secondary antibodies diluted 1:1000 in blocking solution for 1 h at RT. After final washing with PBS, coverslips were mounted with Fluoromount G or Fluoromount G-DAPI (SBA). For image acquisition, a Leica laser-scanning confocal microscope (Leica TCS-SP5 II) with a 63x objective was used.

### Bacterial expression and purification

Protein expression was conducted using chemically competent *E. coli* Rosetta cells. The cells were grown in auto-induction ZY medium with kanamycin and chloramphenicol for 5 h at 37 °C. The temperature was then decreased to 18 °C and the cells were grown overnight. The cells were harvested by centrifugation and the cell pellet was resuspended in resuspension buffer (200 mM NaCl, 20 mM Tris pH 7.5, 2 mM DTT, 10 mg l-1 lysozyme, 5 mg l-1 DNase I) and subsequently lysed by sonication for 15 min. The lysate was cleared at 56000xg for 45 min and the supernatant was applied to affinity chromatography using a column packed with 2 ml Ni-NTA agarose (NEB). The average incubation time was 1 h. Two washing steps were then performed using 25 ml washing buffer (200 mM NaCl, 20 mM Tris pH 7.5, 20 mM imidazole, 2 mM DTT) for each step. For elution, the Ni-NTA agarose was incubated with 20 ml elution buffer (200 mM NaCl, 20 mM Tris pH 7.5, 400 mM imidazole, 2 mM DTT) for 15 min. The eluted constructs were purified using a Superdex 75 16/60 column (GE Healthcare). The protein-containing fractions were pooled and concentrated using a 30 kDa molecular-weight cut-off concentrator (Millipore). The progress of protein purification was monitored by SDS–PAGE. Protein concentrations were determined by UV absorption with extinction coefficients ε(MPP2) = 52 830 l mol^−1^ cm^−1^ and ε(mCherry-SynCAM1) = 35 870 l mol^−1^ cm^−1^, respectively.

### Isothermal titration calorimetry

ITC experiments were performed on an ITC200 (Microcal) at 25 °C with constant stirring at 500 rpm and a buffer containing 200 mM NaCl, 20 mM Tris-HCl (pH 7.5) and 1 mM DTT. Each experiment comprised a 0.5 μl initial injection, followed by fifteen 2.5 μl injections every 150 seconds. The cell contained 55 μM of the MPP2 PSG module, and 350 μM of mCherry-tagged SynCAM1 peptide or mCherry-tagged mutated SynCAM1 peptide were titrated into the MPP2 PSG solution. Control experiments were carried out titrating mCherry-tagged peptide into ITC buffer under otherwise identical conditions. No heat exchange was detected in the control experiments, confirming that there was appropriate match of buffer conditions with no indication of dilution effects. The thermodynamic parameters were determined using ORIGIN software (v 7.0, Microcal) and fitted by nonlinear least square analysis using a single-site binding model.

## Additional Information

**How to cite this article**: Rademacher, N. *et al*. MPP2 is a postsynaptic MAGUK scaffold protein that links SynCAM1 cell adhesion molecules to core components of the postsynaptic density. *Sci. Rep.* **6**, 35283; doi: 10.1038/srep35283 (2016).

## Supplementary Material

Supplementary Information
