# Supplementary material for: MPP2 is a postsynaptic MAGUK scaffold protein that links SynCAM1 cell adhesion molecules to core components of the postsynaptic density
Source: Sci Rep. 2016 Oct 19;6:35283. doi: 10.1038/srep35283 (PMC5069480; doi:10.1038/srep35283)
Supplement: Supplementary Information [file srep35283-s1.pdf]

MPP2 is a postsynaptic MAGUK scaffold protein that links SynCAM1 cell adhesion molecules to core components of the postsynaptic density

Nils Rademacher+, Bettina Schmerl+, Jennifer A. Lardong, Markus C. Wahl, and Sarah A. Shoichet*

Supplementary Figure S1

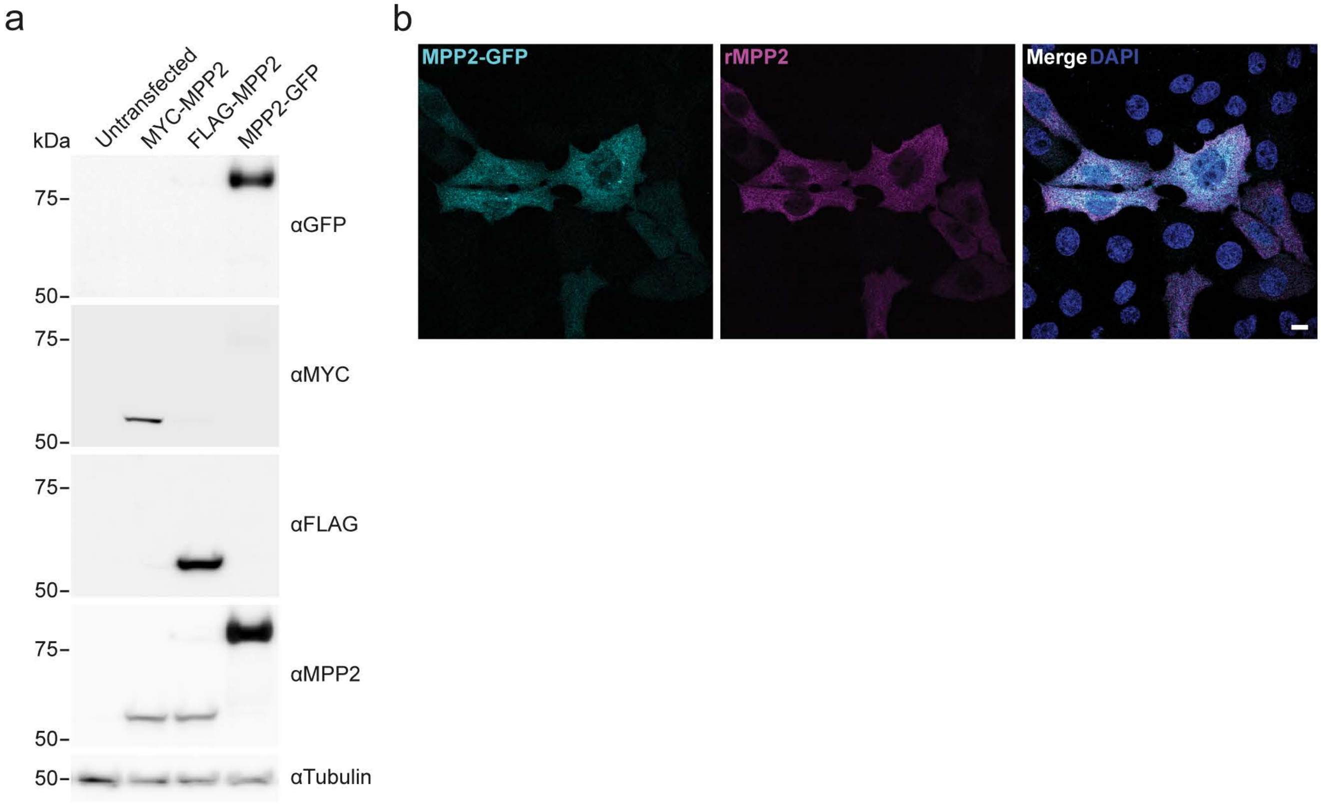

Figure S1 Verification of polyclonal rabbit MPP2 antibody specificity

We validated the specificity of the αMPP2 antibody (rabbit, Abcam) with COS7 cells transiently transfected with differentially tagged expression constructs and analysed in western blot (a). The αMPP2 antibody reliably detects all tagged constructs. In untransfected COS7 cells we observe no signal with rMPP2, also indicating no endogeneous expression of MPP2 in these cells. COS7 cells were transiently transfected with MPP2-GFP and fixed 24hr post-transfection. Immunofluorescence staining with αMPP2 antibody shows a total overlap of GFP signal with αMPP2 staining, whereas untransfected cells (no GFP signal; exclusively DAPI positive) remain unstained (b); scale bar = 5µm.

Supplementary Figure S2

Fig. 1a

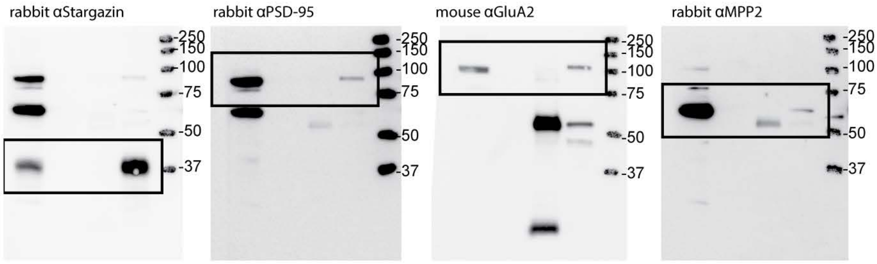

Fig. 2b

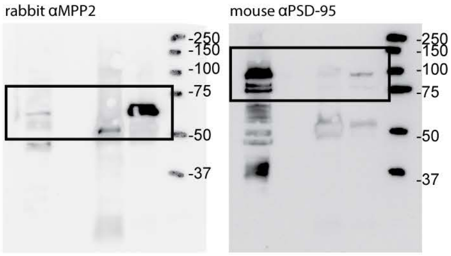

Fig. 2c

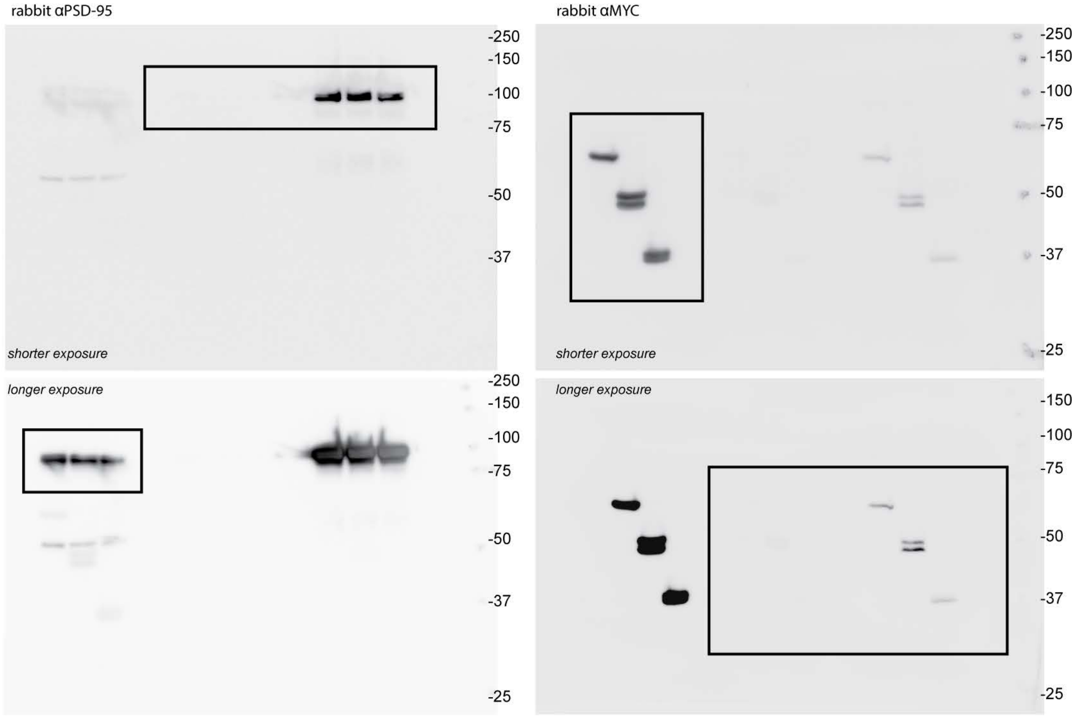

Fig. 2d

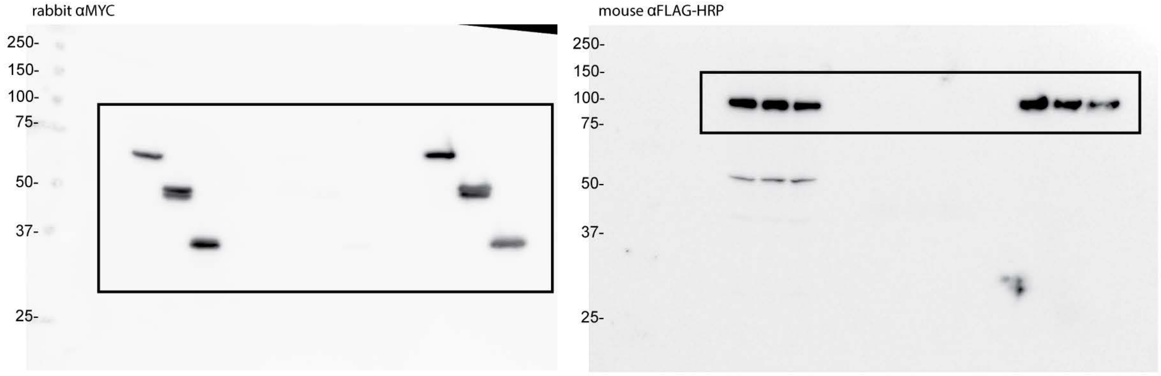

Fig. 2e

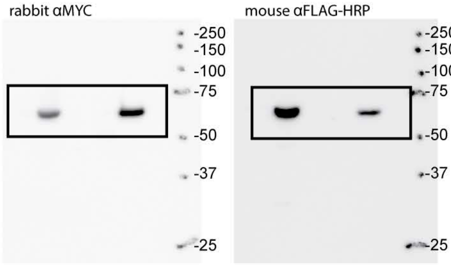

Figure S2 Full length blots of main figures 1 and 2

Uncropped full length blots from main figures as indicated above. Black boxes represent area used in main figure. Used antibodies are given above blot.

Supplementary Figure S3

Fig. 3a (upper panel)

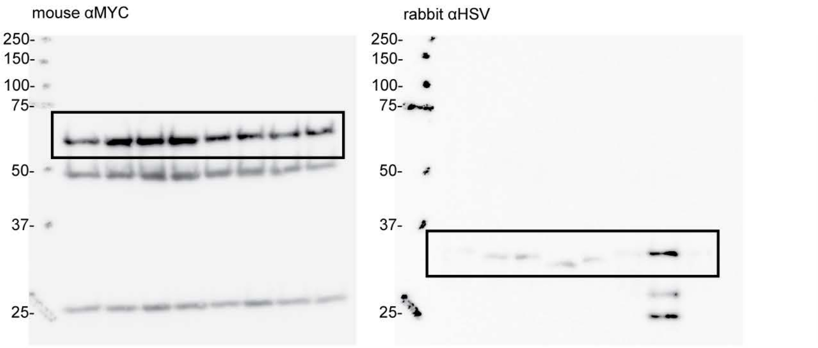

Fig. 3a (lower panel)

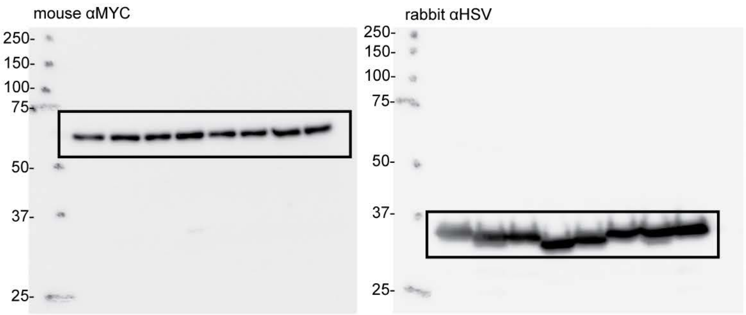

Fig. 3c

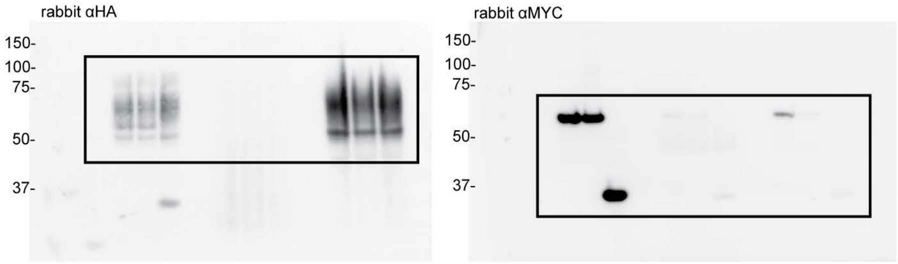

Fig. 3d

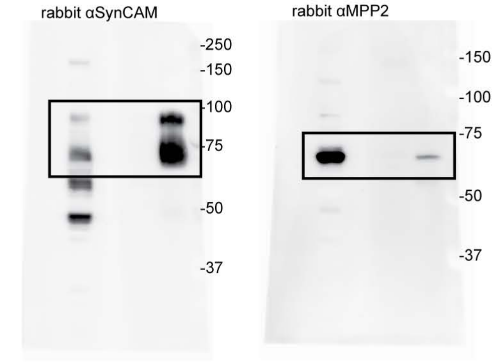

Figure S3 Full length blots of main figure 3

Uncropped full length blots from main figure as indicated above. Black boxes represent area used in main figure. Used antibodies are given above blot.

Table S1: Yeast-Two-Hybrid Screen of mouse MPP2 vs Rat hippocampal cDNA library (Hybrigenics)

Bait	*Mus Musculus* MPP2 PSG Module (AA 119-552) Gene ID: 50997	Clones detected
Prey	*Rattus Norvegicus* SynCAM1/Cadm1/TSLC1/Igsf4 (AA 402-417) Gene ID: 363058	24
	Rattus Norvegicus Dlgap1 (AA 245-399) Gene ID: 65040	11
	Rattus Norvegicus Dlgap1 (AA 28-237) Gene ID: 65040	5
